# Evaluation of the innate immunostimulatory potential of originator and non-originator copies of insulin glargine in an *in vitro* human immune model

**DOI:** 10.1371/journal.pone.0197478

**Published:** 2018-06-06

**Authors:** Ernesto Luna, Pankaj Agrawal, Riyaz Mehta, Maria E. Boone, Charlotte Vernhes, Colombe Denys, Robert Small, Bhaswati Mukherjee, Norbert Tennagels, Stefan Maerten, Donald R. Drake

**Affiliations:** 1 Sanofi Pasteur, Orlando, FL, United States of America; 2 Sanofi, Paris, France; 3 Sanofi-Aventis Deutschland GmbH, Frankfurt, Germany; Medical University of Vienna, AUSTRIA

## Abstract

**Background:**

The manufacture of insulin analogs requires sophisticated production procedures which can lead to differences in the structure, purity, and/or other physiochemical properties of resultant products that can affect their biologic activity. Here, we sought to compare originator and non-originator copies of insulin glargine for innate immune activity and mechanisms leading to differences in these response profiles in an in vitro model of human immunity.

**Methods:**

An endothelial/dendritic cell-based innate immune model was used to study antigen-presenting cell activation, cytokine secretion, and insulin receptor signalling pathways induced by originator and non-originator insulin glargine products. Mechanistic studies included signalling pathway blockade with specific inhibitors, analysis of the products in a Toll-like receptor reporter cell line assay, and natural insulin removal from the products by immunopurification.

**Findings:**

All insulin glargine products elicited at least a minor innate immune response comparable to natural human insulin, but some lots of a non-originator copy product induced the elevated secretion of the cytokines, IL-8 and IL-6. In studies aimed at addressing the mechanisms leading to differential cytokine production by these products, we found (1) the inflammatory response was not mediated by bacterial contaminants, (2) the innate response was driven by the native insulin receptor through the MAPK pathway, and (3) the removal of insulin glargine significantly reduced their capacity to induce innate activity. No evidence of product aggregates was detected, though the presence of some high molecular weight proteins argues for the presence of insulin glargine dimers or others contaminants in these products.

**Conclusion:**

The data presented here suggests some non-originator insulin glargine product lots drive heightened in vitro human innate activity and provides preliminary evidence that changes in the biochemical composition of non-originator insulin glargine products (dimers, impurities) might be responsible for their greater immunostimulatory potential.

## Introduction

Insulin glargine is an analog of the native insulin peptide hormone that has a well-established role in regulating carbohydrate metabolism via its ability to increase glucose transport across cell membranes, enhance cellular glycolysis, and trigger glycogen synthesis in various cell types [1]. It is perhaps less well appreciated that insulin glargine, like any peptide, can also affect the immune system: Given the ubiquitous expression of insulin receptors (IRs) on immunocytes that will interact with insulin glargine, insulin glargine can modulate a variety of immune processes, such as chemotaxis, phagocytosis, and chemokine/cytokine production [2,3].

The current day manufacture of insulin analogs requires sophisticated production procedures; slight distinctions in these processes can lead to differences in the structure (protein aggregation and/or denaturation), purity (contamination with endotoxins or other bacterial products), and/or other characteristics of these products [4,5]. It should be anticipated that variations in the biochemical and/or physiochemical properties of manufactured insulin glargines can affect not only their metabolic activity, but also their capacity to interact with the immune system. Although a specific evaluation of the immunomodulatory effect of insulins destined for human use is not commonly performed, immune analyses could serve as a potentially important aspect of the biological characterization of these products [5,6].

Insulin glargine (LANTUS^®^, Sanofi-Aventis, Paris, France) is a recombinant, long-acting insulin analog for the treatment of diabetes mellitus that was introduced in 2000. Since that time, non-originator copies of insulin glargine produced by various manufacturers have been introduced in several countries. All of these insulin glargine products, including the originator LANTUS^®^ insulin glargine product, have not previously been examined for their ability to modulate human immune activity in in vitro studies. Consistent with regulatory guidelines recommending the use of the most precise methodology available to assess immunogenicity [4,7,8], we used the human immune Modular IMmune In vitro Construct (MIMIC^®^), a system developed and used to detect subtle differences in immune activation between biologics (monoclonal antibodies) [9], vaccines (Toll-like receptor [TLR] agonists) [10], and branded and US-generic enoxaparins [11], to examine immune profiles of insulin glargines. In this current study, we directly compared the innate immune potential of originator and non-originator insulin glargines and investigated mechanisms to explain differential immunomodulatory effects observed between these products.

## Methods

### Drug formulations

Non-originator insulin glargines were purchased from local pharmacies. Some batches of originator insulin glargine were obtained from internal sources, while others were purchased from local pharmacies to assess potential changes in the product during storage and/or shipment. No differences were observed between batches purchased from the pharmacy and those stored internally until the time of analysis. Most of the products were used in experiments before their expiration dates; for Basalog lots G030013 and R130007 and Glaritus lot DL10436, experiments were performed using expired products. See Table 1 for additional details.

**Table 1:** Insulin glargines employed in this study.

Manufacturer	Batch No.	Expiration date	Time period cell-based assay	Time period HPLC analysis	Content m-cresol (mg/mL)	Content insulin glargine (IU/mL)	Largest single related protein (%)	Sum of related proteins (%)	HMWP (%)
**Basalin (Gan&Lee), China**	12101211	05/2013	08/2013–03/2014	09/2011	2.64	98.7	0.2	0.9	0.3
	12111009	03/2014	08/2013–03/2014	10/2012	2.68	101.6	0.2	1	0.3
**Basalog (Biocon), India**	G030013	04/2012	08/2013–03/2014	05/2011	2.67	98.1	0.2	0.8	0.2
	G040099	12/2013	08/2013–03/2014	10/2012	2.67	100.2	0.2	1	0.1
	G040122	09/2014	08/2013–03/2014	08/2013	2.68	101.9	0.1	0.6	0.1
	R130007	09//2013	08/2013–03/2014	07/2013	2.69	95.8	0.2	0.9	0.2
**Glaritus (Wockhardt), India**	DL10436	03//2013	08/2013–06/2015	05/2011	2.53	95.5	0.3	1.4	0.4
	DM11832	06//2015	08/2013–06/2015	10/2012	2.61	103.3	0.7	3.3	0.1
	DN10870	07/2015	08/2013–06/2015	07/2013	2.66	100	0.9	2.2	0.1
	DN10896	07/2015	08/2013–06/2015	07/2013	2.65	98.3	0.9	1.9	0.1
**Bonglixan (Landsteiner), Mexico**	LPTP12E092	6/14/2014	8/2013–3/2014	3/2014	2.68	99.9	0.2	0.9	0.1
**Lantus (Sanofi)**	0F109	10/12/2012	8/2013–3/2014	6/2013	2.71	100.1	0.2	0.7	0.1
	0F004	1/13/2013	8/2013–3/2014	9/2011	2.62	97.4	0.3	0.9	0.1
	2F406	4/15/2015	8/2013–6/2015	10/2012	2.69	99.0	0.2	0.6	<0.05
	3F080	6/15/2015	8/2013–6/2015	5/2014	2.71	100.0	0.1	0.6	<0.05

### PBMC preparation

Apheresis blood products were collected from donors at the OneBlood blood bank (Orlando, FL, USA). The study protocol and donor program were reviewed and approved by Chesapeake Research Review Inc. (Columbia, MD, USA). At the time of the collection, peripheral blood mononuclear cells (PBMCs) from healthy donors were enriched by Ficoll density gradient separation and cryopreserved in DMSO-containing freezing media according to standard laboratory procedures. PBMCs were chosen at random from our pool for inclusion in each experiment [12].

### MIMIC^®^ PTE assay

The MIMIC^®^ PTE construct was assembled on a robotic line using published methods [10,13,14]. Briefly, endothelial cells were grown to confluence atop a collagen matrix (Advanced Biomatrix, San Diego, CA, USA). Thereafter, donor PBMCs prepared from frozen stocks were applied to the assay wells. After a 90-minute incubation, non-migrated cells were washed away, and each insulin product (Table 1) was added at a concentration of 5.0 or 0.5 U/ml (30 or 3 nM). Human Insulin (Insuman Rapid^®^, 100 IU/ml, batch 3F190A, Sanofi-Aventis, Frankfurt am Main, Germany) was added as a control for insulin biological effects. A mixture of 50 ng/mL lipopolysaccharide and 10 μg/mL R848 (InvivoGen, San Diego, CA, USA) was used as a positive control in these assays. The culture supernatant were harvested after 48 hours and analyzed for cytokines/chemokines via a multiplex assay.

### Signaling pathway experimental design

MIMIC^®^ PTE cultures were incubated with the following agents immediately following PBMC application: 10 μg/ml anti-IGF-IR/IGF-1R antibody (1H7) (Thermo Fisher Scientific, Waltham, MA, USA),10 nM insulin receptor AB antagonistic peptide S961 [15],10 nM rapamycin (mTOR inhibitor, InvivoGen) or 25 nM PD98059 (MEK1 and MEK2 Inhibitor, InvivoGen). (All blocking reagents were adapted from published protocols [16–18]). One hour later, 5 U/ml Glaritus, Basalog, or originator insulin glargine were added to the wells and incubated for 48 hours at 37°C and 5% CO_2_. Thereafter, the culture supernatants were harvested and analyzed for secreted cytokines by multiplex assay.

In some experiments, insulin glargine was removed from the formulation prior to this analysis. Briefly, insulin glargine (175 μg/mL) was incubated with 5 μg/ml anti-insulin mAb (Cell Signaling Technology, Danvers, MA, USA) for 24 hours at 4°C. The reaction mix was then passed through an Amicon Ultra 30 kDa cut-off filter (Millipore, Billerica, MA, USA) to retain the insulin glargine. The filtrate was examined by SDS-PAGE to confirm the insulin glargine was removed from the product.

### Cytokine/Chemokine analysis

MIMIC^®^ culture supernatants were analyzed using a Milliplex^®^ human 16-plex multi-cytokine detection system (Millipore), per the manufacturer’s protocol. The kit includes VEGF, IFN-α2, IFNγ, IL-1β, IL-1Ra, IL-6, IL-8, IL-10, IL-12, IL-12, IP-10, MCP-1, MIP-1α, MIP-1β, RANTES, and TNF-α. Analyte concentrations were calculated based on relevant standard curves using the Bio-Plex manager software.

### TLR reporter cell line assay

THP1-XBlue™-CD14 (InvivoGen) cell analysis was performed following the manufacturer’s protocol. The cells were authenticated and confirmed to be mycoplasma free by the manufacturer. Briefly, the cells were treated with all insulin glargine compounds at a dose of 5 U/ml (30 nM) for 18–24 hours. Thereafter, the cells were incubated with QUANTI-Blue™ and SEAP levels were measured at an absorbance of 622 nm on a Bio-Tek Synergy HT multiwell reader using KC4 software (Bio-Tek Instruments, Winooski, VT, USA).

### Native PAGE assay

500 ng of each insulin glargine was analyzed in a 3–12% gradient Bis-Tris Native-PAGE under non-reducing conditions followed by silver staining (ProteoSilver™ Silver Stain Kit; Sigma-Aldrich, St. Louis, MO, USA). Molecular markers were used as size standards. Images were taken with a Kodak GL 1500 Imaging system.

### Flow cytometry

MIMIC^®^ PTE-derived cells were washed with PBS and stained with Live-Dead Aqua (InvitroGen, Carlsbad, CA, USA) for 20 minutes on ice. After washing and IgG-Fc blocking (mouse IgG1; Sigma-Aldrich), the cells were incubated with a cocktail of fluorochrome-labeled mAbs specific for non-myeloid lineage cells and the immune markers CD3, CD19, CD14, HLA-DR, CD86, and CD25 (BD Biosciences, San Jose, CA, USA). Thereafter, the cells were washed with buffered media and acquired on a BD Fortessa flow cytometer equipped with BD FACS Diva software (BD Biosciences). Data analysis was performed using FlowJo software (Tree Star, Ashland, OR, USA).

### RP-HPLC and SEC analyses

A determination of the content of insulin glargine and related proteins in the test formulations was made using reverse-phase (RP) high-performance liquid chromatography (HPLC). High-molecular weight proteins (HMWPs) were examined by size-exclusion chromatography (SEC). RP-HPLC and SEC were performed on the basis of the method described in USP39 NF 34 [19].

### Statistical analyses

Statistical analyses and graphs were prepared using GraphPad InStat version 5.00 (GraphPad Software, San Diego, CA, USA) and SAS version 9.4 (SAS, Cary, NC, USA). Data were presented using mean and SEM, paired t-test, and ANOVA depending on the number of variables analysed. For the randomized complete block analysis, IL-6 and IL-8 data from 12 donors were divided and the assignment of treatments was within a donor, giving a randomized complete block design that was blocked on donor variability. This is similar to the study design typically used in pharmacokinetic studies of drugs [20,21]. Thereafter, analysis of variance was performed and Tukey’s honestly significant differences (HSD) were employed to determine statistical significance. To make the data more nearly normally distributed, logarithms of the responses were used to perform these analyses. The pairwise comparisons were done in the log-scale and the 95% Confidence intervals (CIs) were calculated in the log scale. The final CIs were built by back-transforming to the original scale. The differences of the logarithms became ratios of the geometric mean value ratios (GMVRs). The multiple comparisons were not corrected for multiplicity since the effects were planned and, further, the results (p-values) were overwhelming in the significant case, which obviated the need for correction. Only p values <0.05 were considered statistically significant.

## Results

### MIMIC^®^ PTE overview

The immunomodulatory effects of originator and non-originator copy insulin glargines were compared in the MIMIC^®^ innate (PTE) model. The PTE is a construct that permits the interrogation of innate immunity and recapitulates the derivation of dendritic cells (DCs) under conditions designed to replicate the physiologic migration of monocytes through the vasculature into tissue sites, where they differentiate into DCs. The current format of the construct is built on a foundation of research by the groups of Muller and Randolph [14] to develop a tissue construct housing primary human umbilical vascular endothelial cells (HUVECs) that promotes the differentiation of blood monocytes into APCs in the absence of exogenous growth factors or cytokines. S1 Fig provides an illustration of the PTE construct and distinctions between this process and the traditional protocol used to generate cytokine-derived dendritic cells that are ubiquitously used in research and clinical studies [22].

### Insulin glargine products trigger minimal changes in MIMIC^®^ PTE cell viability and phenotype

All insulin glargine products were tested for their ability to alter the viability and/or activation status of 12 donor PBMC samples in 48-hour MIMIC^®^ PTE assays. Independent of treatment dose (30 nM/L or 3 nM/L), all insulin glargine products triggered essentially no reduction in viability of the PTE-derived cells (S2 Fig and S3 Fig, upper panel), which demonstrates both doses were well tolerated in the assay system and triggered little/no immunocytotoxicity. Likewise, the harvested cells showed little/no alteration in their expression of a variety of cell surface markers, such as HLA-DR, CD86, and CD83, that would have been indicative of the activation/maturation of the PTE-derived APCs (data not shown). This suggests none of the insulin glargine products have the potential to modulate DC activity over a broad dose range, which is a desirable trait in a therapeutic drug since APCs serve to drive and amplify adaptive immunity. It should be noted a positive assay control − a combination of the TLR agonists, LPS and R848 –triggered an expected and statistically significant decrease in cell viability in the system whereas a reference human insulin (Insuman) control triggered little change in cell viability or phenotype (S2 Fig).

### Originator and insulin glargine copies induce differential chemokine/cytokine responses in the MIMIC^®^ PTE construct

**Fig 1:**
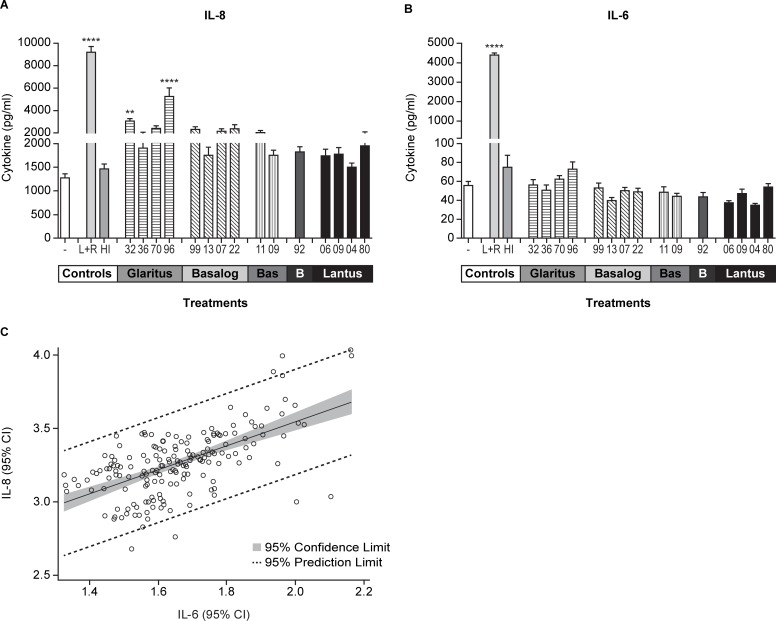
Insulin glargine (Glaritus) lots 32 and 96 induce heightened innate activity in the MIMIC^®^ PTE. MIMIC^®^ PTE cultures were treated with different batches of insulin glargines at 30 nM (5 U/ml) for 48 hours. Thereafter, the culture supernatant were collected and evaluated for the secretion of different cytokines by multiplex assay. The plotted values represent mean ± SEM (pg/ml) of IL-8 secretion (**A**) and IL-6 secretion (**B**) for 12–14 donors. (**C**) Graphical representation of Pearson correlation analysis using confidence intervals (CI) of IL-8 and IL-6 secretion induced by all insulin glargine products. **, p<0.01; ****, p<0.001; -, No treatment; L+R, LPS+R848; Bas, Basalin; B, Bonglixan; HI, Human Insulin (Insuman). Lot numbers represent the last two digits of the lot numbers shown in Table 1.

Despite not observing APC phenotypic changes in the MIMIC^®^ PTE, we considered the possibility that the insulin glargines might have triggered the production of inflammatory cytokines/chemokines in the construct during the 48-hour treatment period. In a 16-plex analyte analysis of the culture supernatants, we found the test agents failed to induce changes in expression of most chemokines/cytokines included in the assay (data not shown), with the exception of the pro-inflammatory factors, IL-8 and IL-6. Though all insulin glargines stimulated at least a minimal increase in the expression of IL-8, as compared with the negative (no treatment) control (Fig 1A), it is notable that Glaritus lots 32 (G32) and 96 (G96) triggered IL-8 production that was approximately 2–3-fold greater than any other insulin glargine lots in multiple experiments and donors (Fig 1A). Though the differential was not as great, there was a trend of increased IL-6 following insulin glargine treatment, particularly in cultures incubated with 30 nM G96. (Fig 1B). Paired confidence interval analyses showed G32 and G96 induced significantly more IL-8 and IL-6 secretion (p<0.001) than the Lantus lots, L04 and L06 (Table 2). Additionally, it is notable that across all observations with all insulin glargine products (n = 12 donors), IL-8 and IL-6 secretion were positively and significantly correlated (R = 0.71, p < .0001) (Fig 1C, upper panel). Since we did not detect significant changes in cell viability and IL-8/IL-6 secretion at the lower (3 nM) treatment dose (S3 Fig), we used the 30 nM dose for all subsequent studies.

**Table 2:** Statistical analysis of IL-8 and IL-6 secretion: Glaritus versus Lantus.

	Parameter	Mean (SE) Difference: Glaritus-Lantus	Mean Difference: 95% CI		P value	Estimated Ratio^a^ (LL, UL)
**IL-8 secretion**	G32-LAN06	0.333 (0.133)	0.071	0.595	0.0130	1.40 (1.07, 1.81)
	G32-LAN04	0.376 (0.133)	0.114	0.638	0.0052	1.46 (1.12, 1.89)
	G96-LAN06	0.654 (0.133)	0.392	0.916	<0.0001	1.92 (1.48, 2.50)
	G96-LAN04	0.696 (0.133)	0.434	0.958	<0.0001	2.01 (1.54, 2.61)
**IL-6 secretion**	G32-LAN06	0.550 (0.147)	0.261	0.840	0.0002	1.73 (1.30, 2.32)
	G32-LAN04	0.686 (0.147)	0.396	0.976	<0.0001	1.99 (1.49, 2.65)
	G96-LAN06	0.914 (0.147)	0.6243	1.204	<0.0001	2.49 (1.87, 3.33)
	G96-LAN04	1.050 (0.147)	0.7599	1.340	<0.0001	2.86 (2.14, 3.82)

^a^Glaritus/Lantus. CI, confidence interval; LL, lower limit; UL, upper limit.

### Insulin glargine-induced IL-8 secretion in the PTE construct is immune cell-dependent

Given the insulin glargine-induced inflammatory response was narrowly focused on IL-8 and IL-6, and these factors can be secreted by either monocytic populations or endothelial cells [23], we questioned whether the heightened production of innate factors by G32 and G96 might be driven by endothelial rather than immune cells. To test this theory, MIMIC^®^ PTE constructs established with endothelial cells but without the PBMC application were treated with the non-originator insulin glargine formulations for 48 hours and then the culture supernatants were examined by multiplex chemokine/cytokine analysis (S4 Fig). Since the products failed to trigger any change in IL-8 secretion in endothelial cell-only PTE constructs, we concluded the response requires the involvement of immune cells. Additional experiments would be needed to determine whether insulin or insulin analogs acts directly on immune cells to produce chemokines or whether an interaction between immunocytes and endothelial cells is needed to trigger IL-8/IL-6 secretion in the system.

### Cytokine/Chemokine responses in MIMIC^®^ PTE assays

Considering the insulin glargine products used in the assays described above have some portion of their peptide sequence in common with native insulin, we hypothesized the differential cytokine response induced by G96 and G32 derived from another component of the tested formulations rather than the insulin glargine molecule itself. Since the nature of the immunomodulatory signal(s) was pro-inflammatory, we considered the possibility that the Glaritus insulin glargine formulations were contaminated with one or more bacterial components capable of triggering a TLR-mediated inflammatory response. However, when evaluated for a variety of TLR agonists/bacterial contaminants using the THP-1 XBlue TLR-sensitive reporter cell line model, all of the insulin glargine products were found to be negative for the presence of any bacterial component (S5 Fig).

**Fig 2:**
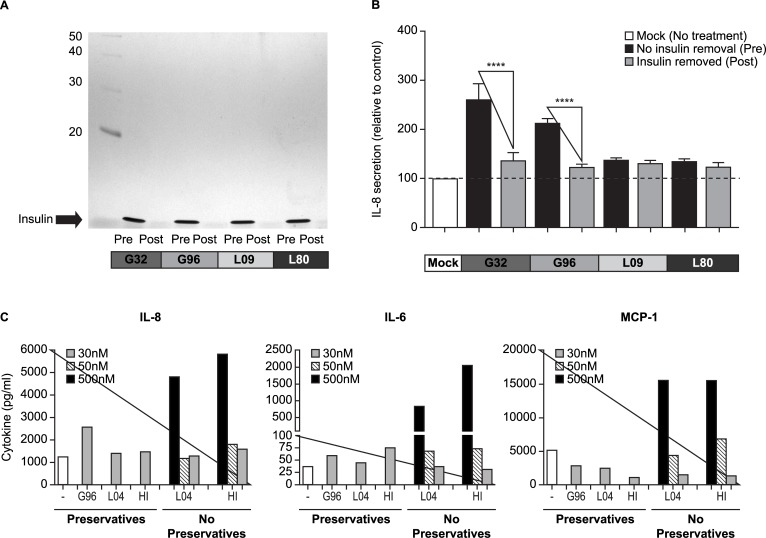
Removal of insulin glargine from Glaritus lots 32 and 96 abrogates innate immune activity induced by these formulations. Insulin glargines were incubated overnight with the L6B10 anti-insulin mAb and then processed through a 30 kDa filter. (**A**) Pre- and post-filtration samples were analyzed by a 12% reducing SDS-PAGE gel; the insulin glargine band is observed at 6–10 kDa. (**B**) Pre- and post-filtration samples were analyzed by 48-hour MIMIC^®^ PTE assay, then multiplex assay for IL-8 production. Data from 19 healthy donors were plotted as % IL-8 secretion over no-treatment control. (**C**) MIMIC^®^ PTE cultures were treated with preservative and non-preservative insulins at doses of 30, 50, and 500 nM and then examined for IL-8, IL-6, and MCP-1 secretion by multiplex assay. Data is plotted as mean ± SEM (pg/ml) from 8–13 healthy donors. ****, p <0.001.

In order to determine whether a preservative and/or other unspecific component in G32 and G96 led to the elevated cytokine response in MIMIC^®^ PTE assays, we used an anti-insulin mAb to remove the insulin glargine from lots G32 and G96 and originator lots L09 and L80 and then tested the insulin glargine-positive and -negative fractions for immunological activity in the in vitro assay. As can be seen in Fig 2A, which shows an SDS-PAGE analysis of the untouched and purified products, we successfully removed a majority of the insulin glargine molecules from the formulations with this immunopurification technique. In a hypothesis that was consistent with published studies [24], we anticipated the cytokine responses were likely driven by non-active components, i.e., the insulin glargine-negative fractions. However, the results of this study demonstrated the strong IL-8 response was, in fact, induced by the insulin glargine-containing fractions of G32 and G96 (Fig 2B, paired t-test, p<0.001). This suggests the insulin glargine protein itself in the G32 and G96 formulations was responsible for the elevated cytokine response observed in MIMIC^®^ PTE cultures. As anticipated based on earlier results showing that the originator product (L09 and L80) did not elicit a significant IL-8 response in the MIMIC^®^ PTE, we found no differential in IL-8 response above the no-treatment control in either the insulin glargine-negative or -positive fractions derived from these products. These observations, which suggest insulin glargine protein directly triggers cytokine/chemokine production in the MIMIC^®^ PTE construct, are further supported by our observation that pure (preservative-free) insulin glargine and human insulin can induce robust cytokine/chemokine responses in a dose-dependent and comparable fashion in MIMIC^®^ PTE assays (Fig 2C). Interestingly, 30 nM of formulated insulins induced more secretion of IL-8 and IL-6 than 50 nM of preservative-free insulins. It suggests some effect of formulation-preservatives perturbing the cultures, probably through aggregations, and enhancing the innate immunity activation.

### IL-8 secretion is triggered via an IR-driven signalling pathway

**Fig 3:**
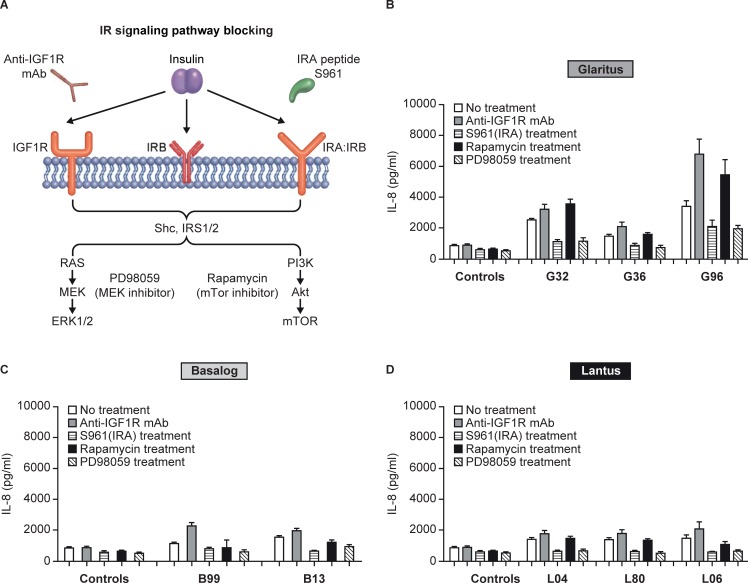
The innate response induced by insulin glargine in MIMIC^®^ PTE assays is largely driven by insulin signaling through the IRA/IRB and MEK pathways. MIMIC^®^ PTE cultures were incubated with the indicated treatments immediately following PBMC application. (S961 is the insulin receptor AB antagonistic peptide.) 1 hour later, 30 nM (5 U/ml) insulin glargines were added to the wells and incubated for 48 hours. Thereafter, the culture supernatants was collected and analyzed for IL-8 secretion by multiplex assay. Data represented as mean ± SEM of 8–12 healthy donors. Lot numbers represent the last two digits of the lot numbers shown in Table 1.

To further confirm whether G32 and G96 exert their immune effects in MIMIC^®^ PTE assays via direct insulin signalling, we performed a series of experiments to examine the specific pathways involved in the IL-6 and IL-8 responses. Native human insulin exerts its effects by binding to the insulin receptor (IR) complex, which results in a kinase transphophorylation that can trigger two distinct intracellular pathways: the phosphatidylinositol-3-kinase (PI3K)-AKT/) pathway and the Ras-mitogen-activated protein kinase (MAPK/MEK) pathway [25]. We perturbed these insulin signalling pathways with a series of blocking agents, as illustrated in Fig 3. Blockade of the IGF-1R with a specific monoclonal Ab or disruption of the PI3K pathway by Rapamycin (which specifically blocks mTOR) did not reduce IL-8 secretion by native insulin or any insulin glargine product, including G32 and G96 (Fig 3). In contrast, blockade of the IRA homodimer and IRA/IRB hybrid receptor with the blocking peptide, S961, or inhibition of the dual-specificity MEK pathway with PD98059, reduced IL-8 secretion by all insulin glargine products, including G32 and G96 (Fig 3). These results suggest that native insulin and all insulin glargine products are capable of driving an IL-8 response via signalling through the IR/MEK pathway and that G32 and G96 are capable of driving a more potent cytokine response through the same signalling pathway.

### Analytical analysis of insulin glargine lots

**Fig 4:**
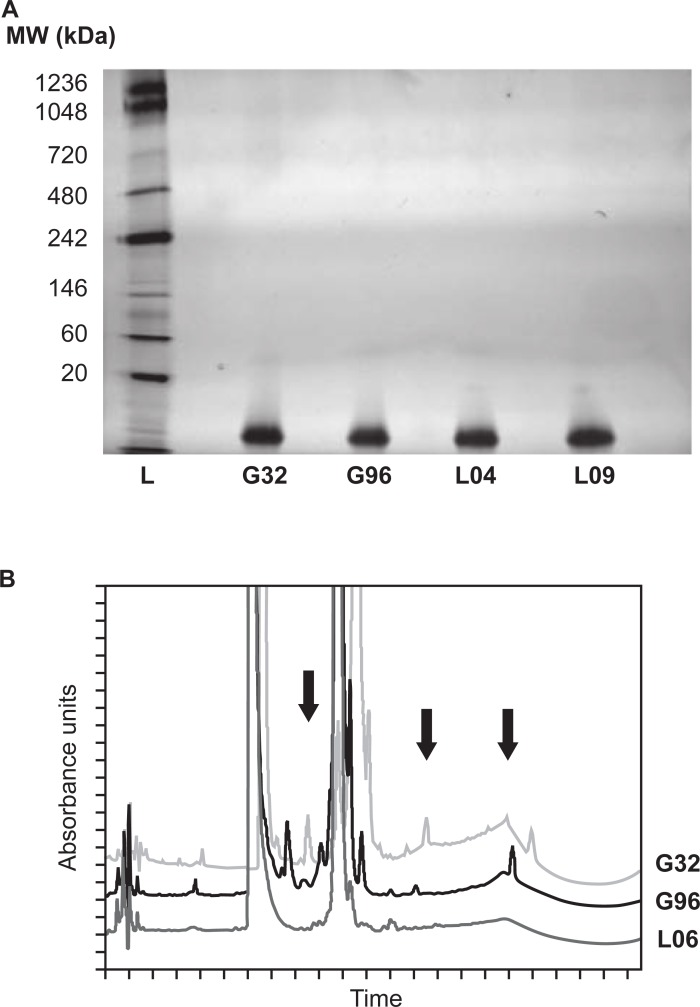
Glaritus lots 32 and 96 show differential by-product profiles by SEC-HPLC analysis. (**A**) 500 ng insulin glargines were analyzed by 3–12% gradient Bis-Tris Native-PAGE under non-reducing conditions followed by silver staining. Molecular markers were used as size standards. Images were taken with a Kodak GL 1500 Imaging system and the insulin glargine band was observed at 6–10 kDa. (**B**) Representative SEC-HPLC analysis of Glaritus lots 32 and 96 compared with the originator lot 06. Arrows indicate the presence of distinct peaks observed between the originator and non-originator insulin glargines.

The above results strongly suggest the elevated cytokine response induced by G32 and G96 are due to signals induced by insulin glargine protein in the formulations, but they do not address why these particular product lots trigger stronger immune activity than other batches of insulin glargines. To investigate this question further, various lots of Glaritus and Lantus were analyzed for the presence of HMWPs or aggregated products via native PAGE and HPLC analyses. Using native PAGE, no high-order complexes or other aggregates were detected in G32 or G96; the observed profile was similar to what was derived from the originator insulin glargine lots, L04 and L09 (Fig 4). However, HPLC revealed extra peaks in G32 and G96 compared with originator insulin glargine L06 (Table 1, Fig 4). Additionally, the profile of G32 and G96 differed slightly from each other. The by-products present in the Glaritus batches potentially resulted from differences in the fermentation and cleavage conditions (e.g., amino acid exchange, trans-peptidation products and miss-cleaved by-products). The nature of other impurities that differ between batches is currently not known; however, an obvious next step would be to test the specific peaks/fractions uncovered by HPLC analysis in the MIMIC^®^ PTE for innate activity and then perform further analytical characterization of these peaks. Unfortunately, there was not sufficient Glaritus material available to perform this study.

## Discussion

Using a novel in vitro human innate immune construct (MIMIC^®^ PTE), we have shown originator and non-originator insulin glargine products have differential capacities to modulate immune cell activity (cytokine production). While the originator insulin glargine generated a minor and consistent immune signal that was comparable to native human insulin, the cytokine response generated by Glaritus was variable from product lot to lot and was often times stronger than the signal generated by the originator insulin glargine product. Further dissection of the Glaritus immunostimulatory effect in this study showed (1) the system required APCs to generate the cytokine secretion, (2) insulin glargine protein itself–rather than preservatives or contaminants–was the driver of the effect, and (3) cytokine production was triggered via the IR and MEK signalling pathways. To the best of our knowledge, this is the first demonstration of such immunological differences between originator and non-originator copies of insulin glargine and suggests differences in the manufacture process can alter the immunobiological properties of insulin glargine products. It should be noted the differences between originator and non-originator insulin glargine products described here are exploratory in nature and without direct clinical significance. Nevertheless, these studies are aligned with a recent study suggesting some batches of insulin glargine biosimilars were capable of triggering allergy due to batch-to-batch variability and accompanying by-products (contaminant antigens) in the formulations [26]. This study suggests it might be useful to do further exploratory studies to address the clinical consequences of immune signals induced by insulin glargine products and to better understand the impact of changes in product formulations in driving these immune signals.

The observation that the cytokine response induced by all insulin glargine products was narrowly focused on IL-8 and IL-6 represents a unique observation in our assay system since most agents capable of generating an immune response typically stimulate the production of a broader array of cytokines and chemokines. This follows well-established dogma suggesting the production of cytokines and chemokines often use common intracellular signalling pathways and intermediates [27]. Nonetheless, this result does seem to align with prior studies showing native human insulin has the capacity to stimulate IL-8 and IL-6 production and few other cytokines/chemokines by monocytes and macrophages [18,28–30]. In fact, in one study, the blockade of the MEK signalling pathway prevented insulin-induced IL-8 (CXCL8) release in primary monocytes [18], which is analogous to our finding that IL-8 secretion in the MIMIC^®^ PTE construct is abrogated when insulin signalling through the IR/MEK pathway is blocked. The unique connection of IL-6 and IL-8 in this study may result from native human insulin and insulin glargine sharing a common (MAPK/ERK) signalling pathway [31], though the exploration of this particular topic was outside the scope of the current study.

Immunopurification techniques were used to generate evidence suggesting the insulin glargine molecule itself, rather than a contaminant in the product, was responsible for the elevated immune activity of the Glaritus 32 and 96 product lots. This conclusion was further supported by our results suggesting (1) these insulin glargines triggered elevated IL-6 and IL-8 but none of the other cytokine/chemokines included in the analysis panel and (2) the responses were blocked with reagents capable of inhibiting insulin signalling pathways. If preservatives and/or biological contaminants were the driver(s) of the heightened immune activity, we would have expected to see a unique pattern of cytokines engaged in response to G32 or G96 stimulation. Though experimental and clinical data suggest aggregation is a major contributor to the immunogenicity of therapeutics [32], native gel analysis suggested the Glaritus product lots did not contain aggregated material. Additional analytical tools would be useful to address the impact of product aggregation on the immune response [33]. Although we did not detect bacterial contaminants in the products via the highly sensitive THP-1 reporter cell line assay, it might be possible that a low level of biological contaminants, such as host cells proteins, nucleic acids, organic or inorganic components, or other molecules (which are defined as innate immune response modulating impurities (IIRMIs)), somehow altered the function of the insulin glargine molecules in these formulations [34]). (Indeed, recent studies suggest minimal levels of IIRMIs can drive innate immune activation [35].) The finding of extra-peaks in Glaritus® lot 96 formulations using HPLC could be representative of this hypothesis. In fact, analytical differences between insulin glargine Lantus^®^ and insulin glargine biosimilars, including Glaritus^®^, have been described [36]. We would have liked to follow up on the analytical characterizations performed here to more formally evaluate what specific physiochemical change in the Glaritus formulations led to the higher immune activity, but we were unable to procure additional vials of these particular product lots.

The results presented in this study highlight a unique approach to evaluating the immunogenicity of insulin glargine products. Whereas researchers often focus on anti-drug antibody (ADA) evaluations as a clinical measure of immunogenicity, we focused on innate immunogenicity as a means to look for subtle immunobiological differences between insulin glargine products from different manufactures. We chose to examine innate rather than adaptive immunity because we believed it would be difficult to detect differences in antibody responses between insulin glargine product lots since this is a biologic known to induce ADA in approximately 50% of patients [35]. Although the exact mechanism leading to the generation of anti-insulin antibodies is not known [37], innate immunity serves as an important driver of adaptive (T and B cells) immune response to drugs [38]. Therefore, understanding how immunogenicity arises at the level of innate immunity may help researchers understand the potential for ADA and design biologics with less immunogenicity potential overall [39]. As such, we believe this analysis demonstrates a role for in vitro immune analysis technologies to provide insights into the immunogenicity potential and consistency of biologic products and would suggest these types of tools should be used alongside other biologic and analytical assays to profile biologics for activity early in the development process.

## Supporting information

S1 Fig Schematic illustration of the MIMIC^®^ PTE construct compared with the traditional approach for generating human DCs in vitro. (**A**) Following PBMC application to the MIMIC^®^ PTE construct, APC differentiation/reverse transmigration occurs during the next 48-hour period. (**B**) The MIMIC^®^ PTE construct is a 2-day process requiring no exogenous factors whereas traditional in vitro human DCs are derived from monocytes cultured in exogenous factors for 7 days.
(DOCX)

S2 Fig Insulin glargines trigger minimal changes in MIMIC^®^ PTE cell viability. **MIMIC**^**®**^ **PTE cultures were treated with different batches of insulin glargines at a dose of 30 nM (5 U/ml).** After a 48-hour culture period, the cells were harvested, stained for viability, and examined by flow cytometry. Data from n = 12 healthy donors was analysed and plotted as mean ± SEM. ****, p<0.001 when comparing the positive control (L+R) with the negative control; B, Bonglixan. Two-digit product lots align with product lots shown in Table 1.
(DOCX)

S3 Fig Low-dose insulin glargine treatment triggers no impact on cell viability and cytokine secretion. MIMIC^®^ PTE cultures were treated with 3 nM (0.5 U/ml) of insulin glargines. After a 48-hour culture period, the cells were harvested, stained for viability, and examined by flow cytometry. The culture supernatants were also collected and evaluated for IL-8 and IL-6 secretion by multiplex assay. Data from 12 healthy donors was plotted as mean ± SEM. ****, p<0.001. B, Bonglixan. Two-digit product lots align with product lots shown in Table 1.
(DOCX)

S4 Fig Endothelial cells are not the major driver of cytokine secretion induced by insulin glargine. Endothelial cell-only MIMIC^®^ PTE cultures were treated with different batches of insulin glargines at a dose of 30 nM (5 U/ml). Culture supernatants were collected after a 48-hour culture period and evaluated for IL-8 secretion using multiplex assay. Data is plotted as mean ± SEM (pg/ml) and includes three independent experiments.
(DOCX)

S5 Fig No bacterial (TLR) contaminates were detected in different lots of insulin glargines. The THP1-XBlue™-CD14 reporter cell line was treated with insulin glargines at a dose of 30 nM (5 U/ml) for 18–24 hours. Thereafter, the cells were incubated with QUANTI-Blue™ and SEAP levels (NFκB activation) were measured at an absorbance of 622 nm. Data represented as mean ± SEM and includes three independent experiments.
(DOCX)
