# Supplementary material for: Evaluation of the innate immunostimulatory potential of originator and non-originator copies of insulin glargine in an in vitro human immune model
Source: PLoS One. 2018 Jun 6;13(6):e0197478. doi: 10.1371/journal.pone.0197478 (PMC5991351; doi:10.1371/journal.pone.0197478)

**S1 Fig. Schematic illustration of the MIMIC^®^ PTE construct compared with the traditional approach for generating human DCs in vitro.** (**A**) Following PBMC application to the MIMIC^®^ PTE construct, APC differentiation/reverse transmigration occurs during the next 48-hour period. (**B**) The MIMIC^®^ PTE construct is a 2-day process requiring no exogenous factors whereas traditional in vitro human DCs are derived from monocytes cultured in exogenous factors for 7 days.


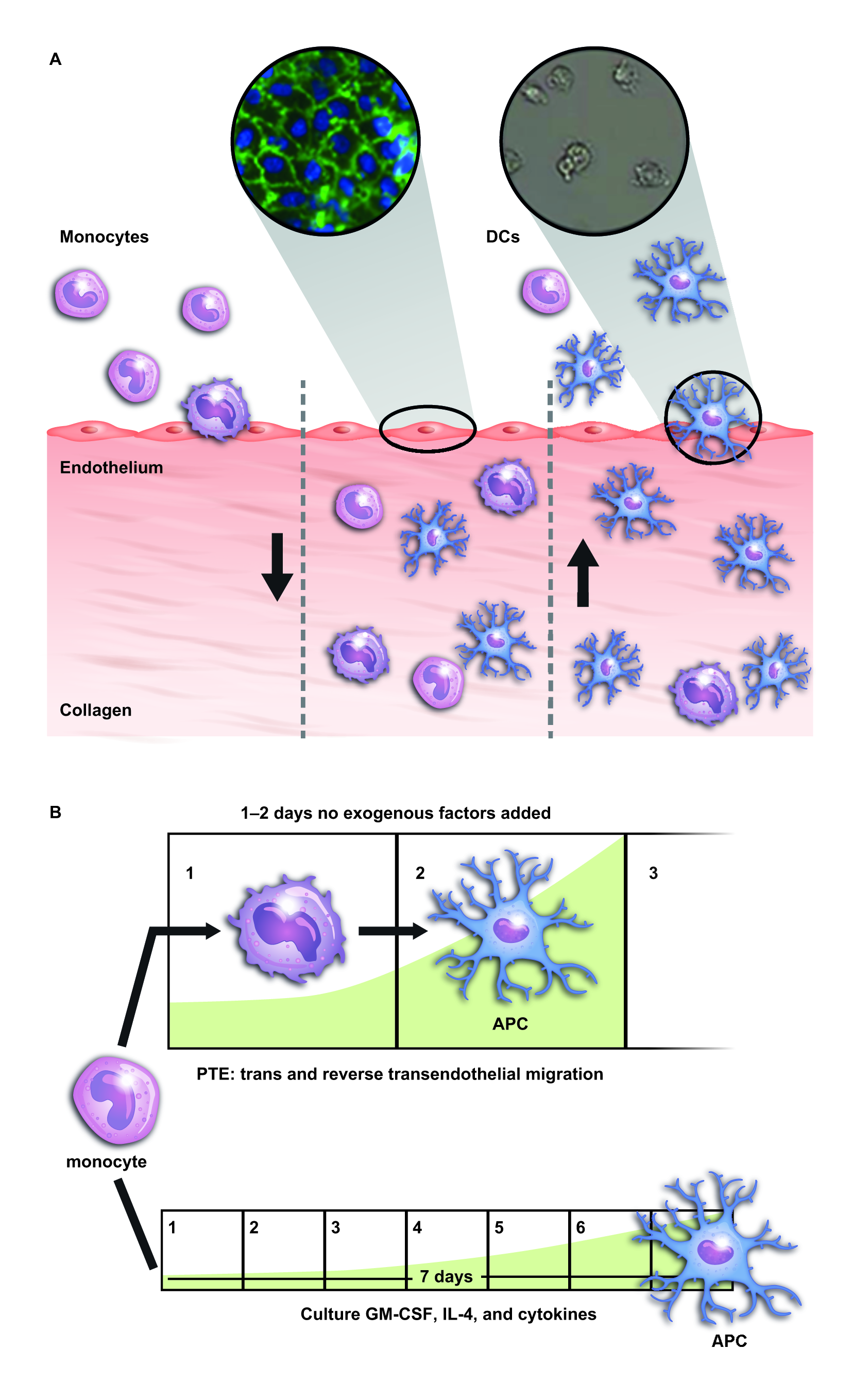

Supplement: S1 Fig — (A) Following PBMC application to the MIMIC® PTE construct, APC differentiation/reverse transmigration occurs during the next 48-hour period. (B) The MIMIC® PTE construct is a 2-day process requiring no exogenous factors whereas traditional in vitro human DCs are derived from monocytes cultured in exogenous factors for 7 days. (DOCX) [file pone.0197478.s001.docx]
