# Supplementary material for: Evaluation of the innate immunostimulatory potential of originator and non-originator copies of insulin glargine in an in vitro human immune model
Source: PLoS One. 2018 Jun 6;13(6):e0197478. doi: 10.1371/journal.pone.0197478 (PMC5991351; doi:10.1371/journal.pone.0197478)

**S2 Fig. Insulin glargines trigger minimal changes in MIMIC^®^ PTE cell viability. MIMIC^®^ PTE cultures were treated with different batches of insulin glargines at a dose of 30 nM (5 U/ml).** After a 48-hour culture period, the cells were harvested, stained for viability, and examined by flow cytometry. Data from n = 12 healthy donors was analysed and plotted as mean ± SEM. ****, p<0.001 when comparing the positive control (L+R) with the negative control; B, Bonglixan. Two-digit product lots align with product lots shown in Table 1.


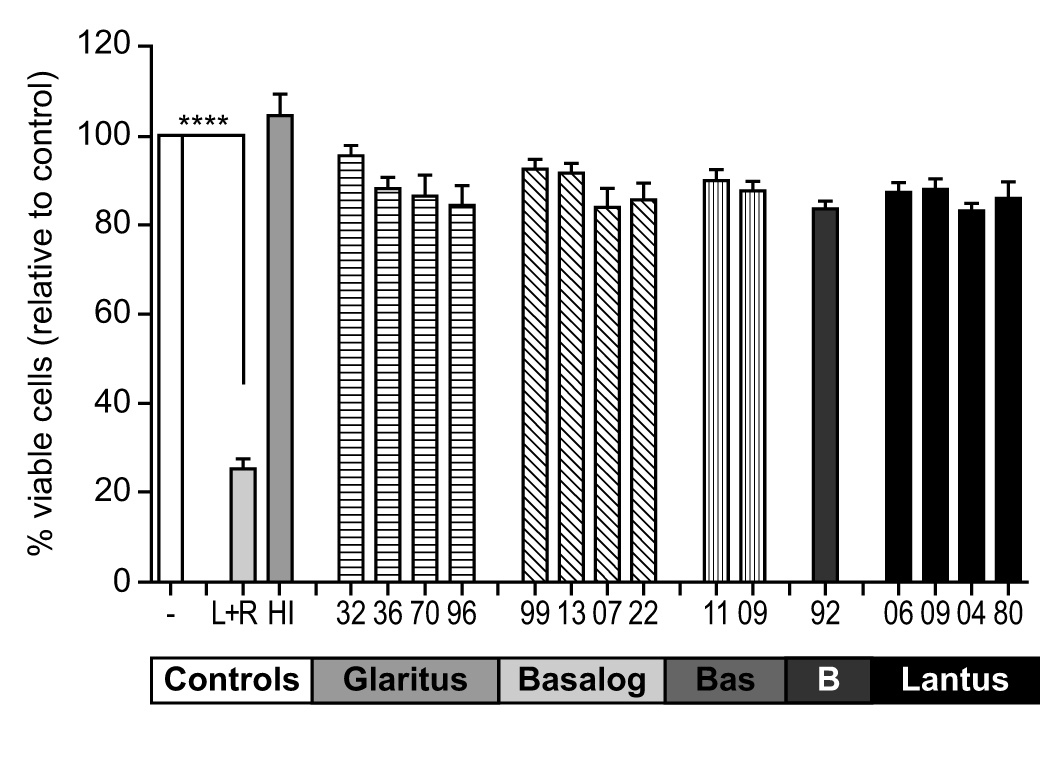

Supplement: S2 Fig — MIMIC® PTE cultures were treated with different batches of insulin glargines at a dose of 30 nM (5 U/ml). After a 48-hour culture period, the cells were harvested, stained for viability, and examined by flow cytometry. Data from n = 12 healthy donors was analysed and plotted as mean ± SEM. ****, p<0.001 when comparing the positive control (L+R) with the negative control; B, Bonglixan. Two-digit product lots align with product lots shown in Table 1. (DOCX) [file pone.0197478.s002.docx]
