# Supplementary material for: Evaluation of the innate immunostimulatory potential of originator and non-originator copies of insulin glargine in an in vitro human immune model
Source: PLoS One. 2018 Jun 6;13(6):e0197478. doi: 10.1371/journal.pone.0197478 (PMC5991351; doi:10.1371/journal.pone.0197478)

**S3 Fig. Low-dose insulin glargine treatment triggers no impact on cell viability and cytokine secretion.** MIMIC^®^ PTE cultures were treated with 3 nM (0.5 U/ml) of insulin glargines. After a 48-hour culture period, the cells were harvested, stained for viability, and examined by flow cytometry. The culture supernatants were also collected and evaluated for IL-8 and IL-6 secretion by multiplex assay. Data from 12 healthy donors was plotted as mean ± SEM. ****, p<0.001. B, Bonglixan. Two-digit product lots align with product lots shown in Table 1.


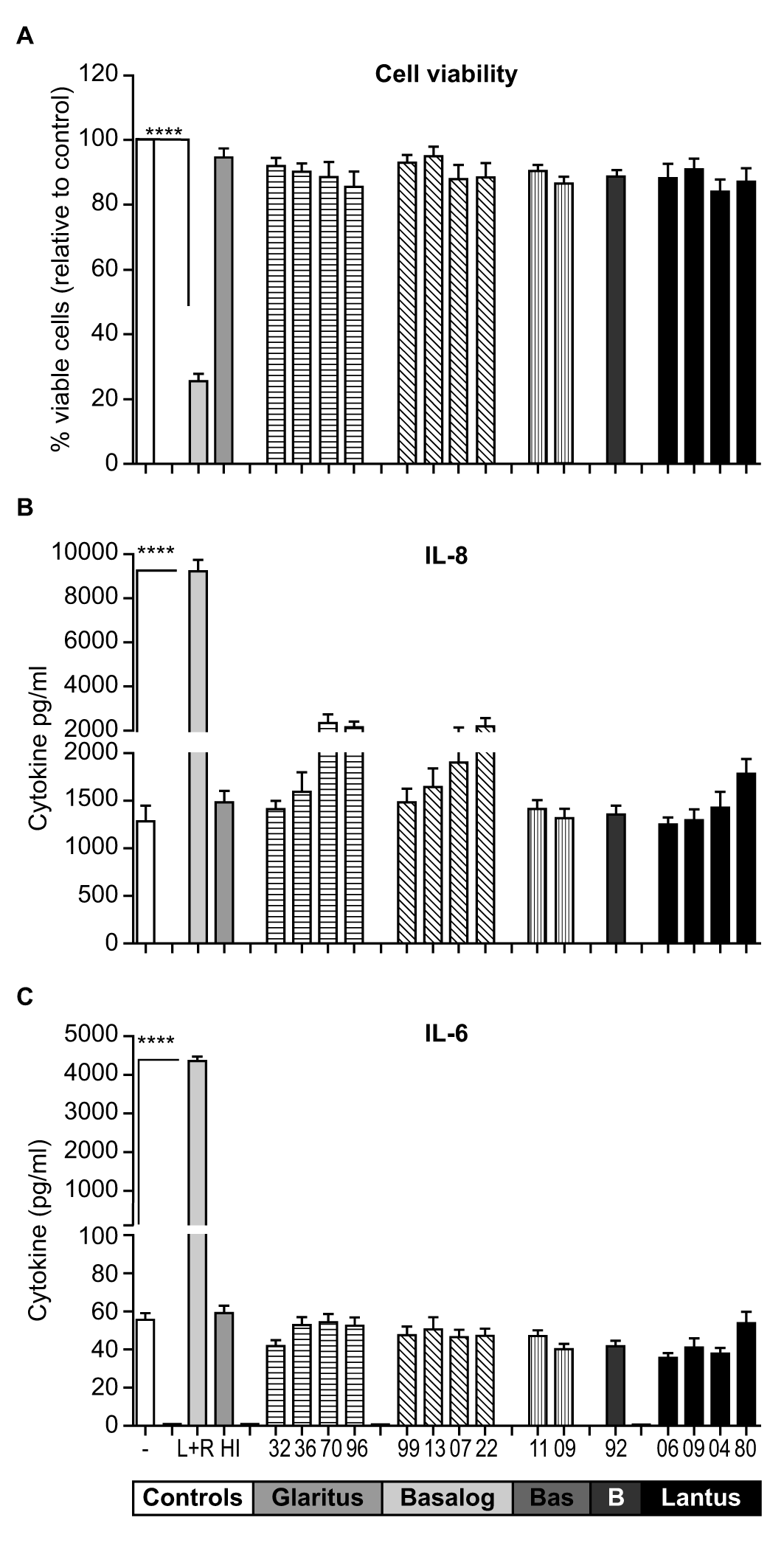

Supplement: S3 Fig — MIMIC® PTE cultures were treated with 3 nM (0.5 U/ml) of insulin glargines. After a 48-hour culture period, the cells were harvested, stained for viability, and examined by flow cytometry. The culture supernatants were also collected and evaluated for IL-8 and IL-6 secretion by multiplex assay. Data from 12 healthy donors was plotted as mean ± SEM. ****, p<0.001. B, Bonglixan. Two-digit product lots align with product lots shown in Table 1. (DOCX) [file pone.0197478.s003.docx]
