# Supplementary material for: Evaluation of the innate immunostimulatory potential of originator and non-originator copies of insulin glargine in an in vitro human immune model
Source: PLoS One. 2018 Jun 6;13(6):e0197478. doi: 10.1371/journal.pone.0197478 (PMC5991351; doi:10.1371/journal.pone.0197478)

**S4 Fig. Endothelial cells are not the major driver of cytokine secretion induced by insulin glargine.** Endothelial cell-only MIMIC^®^ PTE cultures were treated with different batches of insulin glargines at a dose of 30 nM (5 U/ml). Culture supernatants were collected after a 48-hour culture period and evaluated for IL-8 secretion using multiplex assay. Data is plotted as mean ± SEM (pg/ml) and includes three independent experiments.


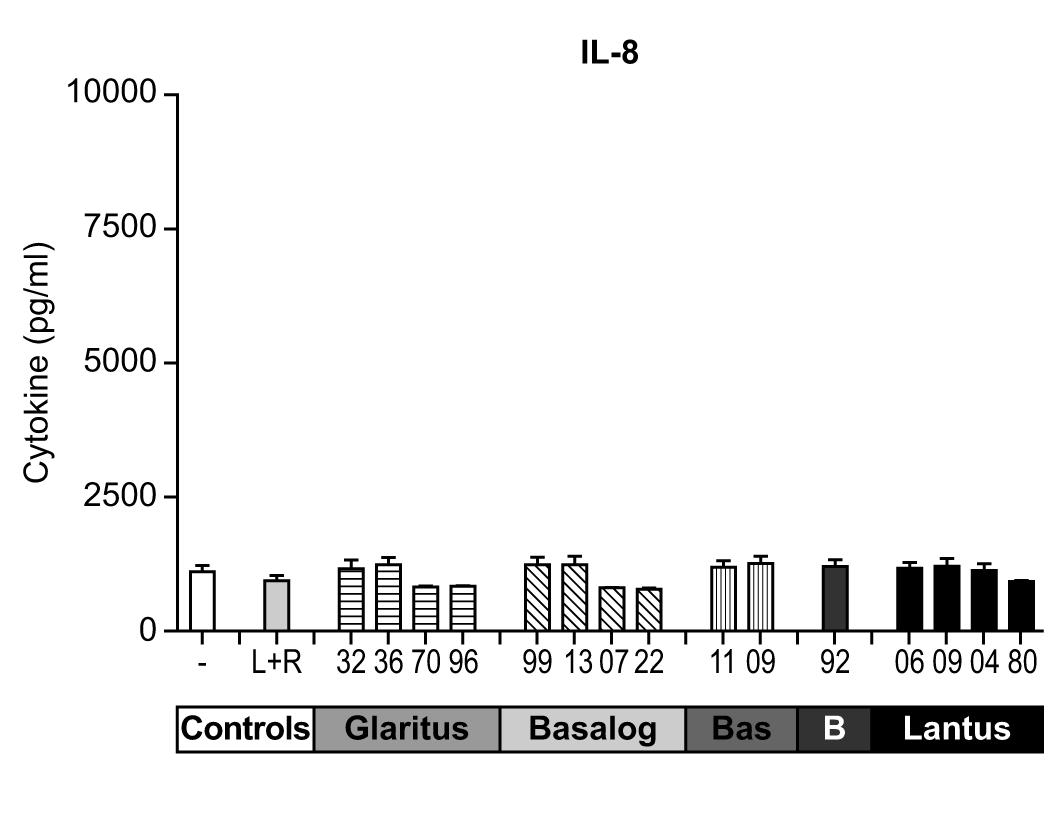

Supplement: S4 Fig — Endothelial cell-only MIMIC® PTE cultures were treated with different batches of insulin glargines at a dose of 30 nM (5 U/ml). Culture supernatants were collected after a 48-hour culture period and evaluated for IL-8 secretion using multiplex assay. Data is plotted as mean ± SEM (pg/ml) and includes three independent experiments. (DOCX) [file pone.0197478.s004.docx]
