# Supplementary material for: Evaluation of the innate immunostimulatory potential of originator and non-originator copies of insulin glargine in an in vitro human immune model
Source: PLoS One. 2018 Jun 6;13(6):e0197478. doi: 10.1371/journal.pone.0197478 (PMC5991351; doi:10.1371/journal.pone.0197478)

**S5 Fig. No bacterial (TLR) contaminates were detected in different lots of insulin glargines.** The THP1-XBlue™-CD14 reporter cell line was treated with insulin glargines at a dose of 30 nM (5 U/ml) for 18–24 hours. Thereafter, the cells were incubated with QUANTI-Blue™ and SEAP levels (NFκB activation) were measured at an absorbance of 622 nm. Data represented as mean ± SEM and includes three independent experiments.


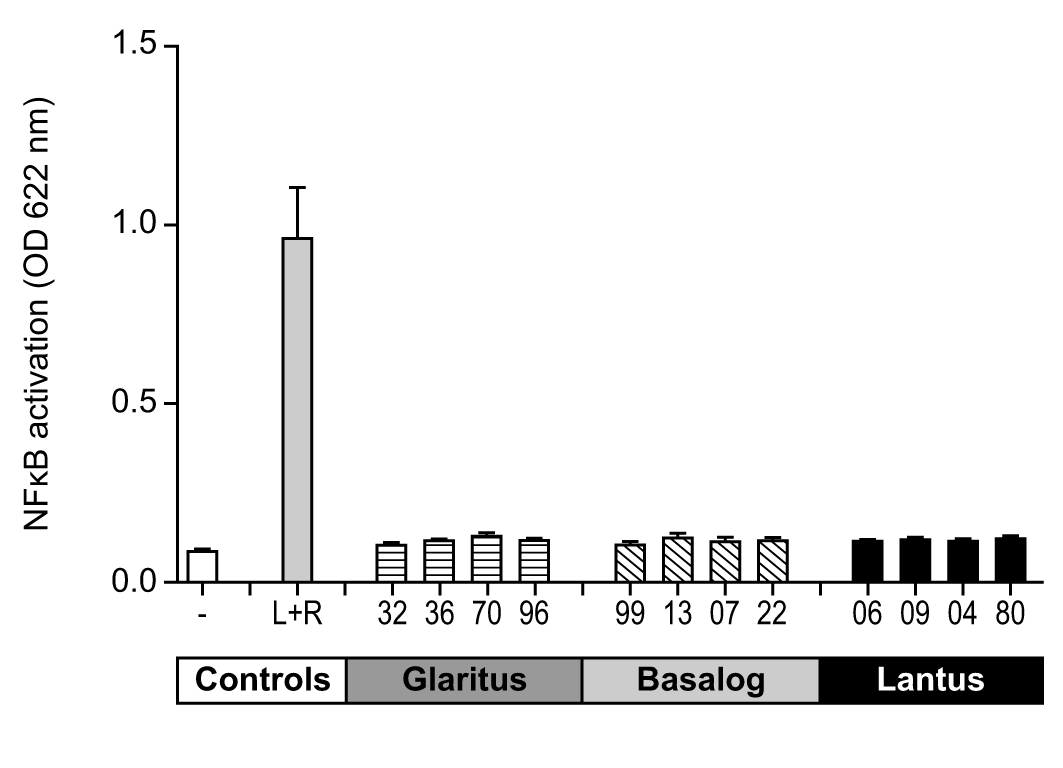

Supplement: S5 Fig — The THP1-XBlue™-CD14 reporter cell line was treated with insulin glargines at a dose of 30 nM (5 U/ml) for 18–24 hours. Thereafter, the cells were incubated with QUANTI-Blue™ and SEAP levels (NFκB activation) were measured at an absorbance of 622 nm. Data represented as mean ± SEM and includes three independent experiments. (DOCX) [file pone.0197478.s005.docx]
